# Impact of combined intermittent fasting and high‐intensity interval training on apoptosis and atrophy signaling in rat fast‐ and slow‐twitch muscles

**DOI:** 10.14814/phy2.16181

**Published:** 2024-08-13

**Authors:** Maria Lua M. Mendonça, Marianna R. Carvalho, Rodrigo B. Romanenghi, Diego S. D. Santos, Wander F. O. Filiú, Luana Urbano Pagan, Katashi Okoshi, Marina P. Okoshi, Rodrigo Juliano Oliveira, Silvio A. Oliveira‐Junior, Paula F. Martinez

**Affiliations:** ^1^ Striated Muscle Study Laboratory Federal University of Mato Grosso do Sul (UFMS) Campo Grande Mato Grosso do Sul Brazil; ^2^ Faculty of Pharmaceutical Sciences, Food and Nutrition Federal University of Mato Grosso do Sul (UFMS) Campo Grande Mato Grosso do Sul Brazil; ^3^ Internal Medicine Department Botucatu Medical School, Sao Paulo State University (UNESP) Botucatu Sao Paulo Brazil; ^4^ Stem Cell, Cell Therapy and Toxicological Genetics Research Centre (CeTroGen), School of Medicine (FAMED) Federal University of Mato Grosso do Sul (UFMS) Campo Grande Mato Grosso do Sul Brazil

**Keywords:** apoptosis inducing factor, physical exercise, skeletal muscle, time restricted feeding

## Abstract

This study aimed to evaluate the influence of combined intermittent fasting (IF) and high‐intensity interval training (HIIT) on morphology, caspase‐independent apoptosis signaling pathway, and myostatin expression in soleus and gastrocnemius (white portion) muscles from healthy rats. Sixty‐day‐old male Wistar rats (*n* = 60) were divided into four groups: control (C), IF, high‐intensity‐interval training (T), and high‐intensity‐interval training and intermittent fasting (T‐IF). The C and T groups received *ad libitum* chow daily; IF and T‐IF received the same standard chow every other day. Animals from T and T‐IF underwent a HIIT protocol five times a week for 12 weeks. IF reduced gastrocnemius mass and increased pro‐apoptotic proteins apoptosis‐inducing factor (AIF) and endonuclease G (EndoG) in soleus and cleaved‐to‐non‐cleaved PARP‐1 ratio and myostatin expression in gastrocnemius white portion. HIIT increased AIF and apoptosis repressor with caspase recruitment domain expression in soleus and cleaved‐to‐total PARP‐1 ratio in gastrocnemius muscle white portion. The combination of IF and HIIT reduced fiber cross‐sectional area in both muscles, increased EndoG and AIF expression, and decreased cleaved‐to‐non‐cleaved PARP‐1 ratio in gastrocnemius muscle white portion. Muscle responses to IF and HIIT are directly impacted by the muscle fiber type composition and are modulated, at least in part, by myostatin and caspase‐independent apoptosis signaling.

## 1 INTRODUCTION

Caloric restriction is characterized by reduced calorie intake without malnutrition (Lee & Longo, 2016). It is often associated with increased longevity in different species and rapid loss of fat mass (Colman et al., 2014; Mercken et al., 2013). Intermittent fasting (IF) is an alternative to traditional calorie restriction and consists of alternating periods of food deprivation and *ad libitum* feeding, generally lasting between 12 and 24 h (Heilbronn et al., 2005). IF has the potential to mitigate metabolic abnormalities and promote several health benefits, such as increasing survival and reducing the development of chronic diseases, including diabetes and cardiovascular diseases (Ahmet et al., 2011; Honjoh et al., 2009).

Despite the potential health benefits, IF is associated with reduced muscle fiber cross‐sectional area in skeletal muscles, with a higher atrophic susceptibility of fast‐twitch fibers (type II) in relation to slow‐twitch fibers (type I) (Almurshed & Grunewald, 2000). Animal studies have shown calorie restriction decreases cross‐sectional area and changes fiber composition in the soleus and extensor digitorum longus muscles (Elashry et al., 2017; Maxwell et al., 1992). In healthy humans, the acute effect of IF is related to activation of pathways that reduce muscle growth (Bonaldo & Sandri, 2013; Sharma et al., 2001).

The mechanism by which IF reduces muscle mass is not yet completely understood. Apoptosis, known as programmed cell death, is an essential process in tissue formation and homeostasis, both in healthy and pathological conditions (Dupont‐Versteegden et al., 2006; Primeau et al., 2002) and may play a role in IF‐induced muscle atrophy. Considering that skeletal muscle fibers are multinucleated, each nucleus regulates and supports gene and protein expression, which is essential in preserving the integrity of a specific region located within the fiber, called the myonuclear region (Allen et al., 1999).Thus, apoptosis in skeletal muscle may also be the result of the selective loss of individual nucleus through a process called “nuclear apoptosis,” instead of entire fiber death (Allen et al., 1999) which can contribute to muscle atrophy.

Apoptotic signaling cascades can be triggered through several pathways (McMillan & Quadrilatero, 2011). These include the caspase‐independent pathway related to sarcoplasmic reticulum stress (Chen et al., 2020; McMillan & Quadrilatero, 2011), with release of apoptosis‐inducing factor (AIF) and endonuclease G (EndoG) from mitochondria, inducing large‐scale DNA fragmentation and apoptosis upon translocation to the nucleus (Candé et al., 2004; Dupont‐Versteegden et al., 2006; van Loo et al., 2001). The mechanisms involved in muscle cell apoptosis also include changes in antiapoptotic proteins, such as the apoptosis repressor with caspase recruitment domain (ARC), and DNA repair proteins, such as Poly (ADP‐ribose) polymerase‐1 (PARP‐1) (Kraus, 2008; McMillan & Quadrilatero, 2011).

Myostatin, a protein in the transforming growth factor family, can also play a role in muscle atrophy (Sharma et al., 2001). Myostatin negatively regulates muscle growth by modulating satellite cells activation and differentiation and hypertrophy pathways (Kvedaras et al., 2019). Myostatin inhibition is associated with increased muscle mass in experimental studies (Wang & McPherron, 2012).

Even though there is evidence showing an association between IF and reduced muscle mass (Almurshed & Grunewald, 2000; Bonaldo & Sandri, 2013), whether physical exercise can attenuate calorie restriction‐induced muscle alterations still needs further investigation. Among the different types of exercises, high‐intensity interval training (HIIT) has become popular due to its time/benefit ratio, which provides rapid results in a shorter daily routine (Blue et al., 2018). This training modality is characterized by periods of high‐intensity exercise (around 90% of VO_2 Max_) alternating with moments of active or passive recovery (Buchheit & Laursen, 2013). HIIT has a positive impact on cardiovascular health and may induce muscle hypertrophy in both healthy and chronic disease individuals (Blue et al., 2018; Estes et al., 2017; Tzanis et al., 2017). However, the effects of HIIT on skeletal muscle during IF still need to be elucidated.

Although it is known that both HIIT and IF promote health benefits, the precise mechanisms underlying their effects on healthy rat skeletal muscle are not completely understood. In this study, we evaluated the impact of combined IF and HIIT on morphology, the caspase‐independent apoptosis signaling pathway, and myostatin expression in healthy rat soleus and gastrocnemius (white portion) muscles.

## 2 METHODS

### 2.1 Animals

**FIGURE 1:**
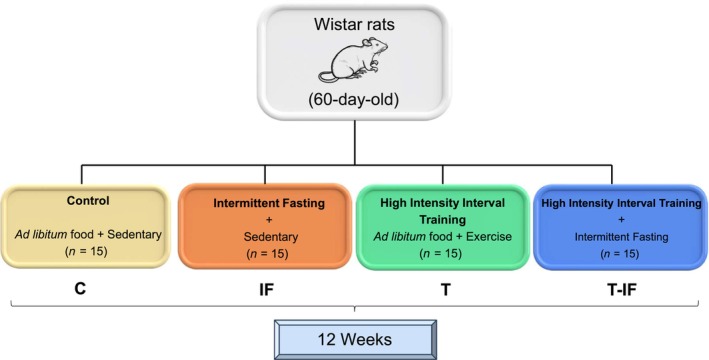
Schematic figure summarizing the experimental design.

Male 60‐day‐old Wistar rats (*n* = 60) were housed (three rats per cage) in a room under standardized light–dark cycles (12:12 h) and temperature (22 ± 2°C). Exercise testing and running protocols were performed during the dark cycle. Rats were distributed into four groups: control (C, *n* = 15), IF (*n* = 15), HIIT (T, *n* = 15) and HIIT combined with intermittent fasting (T‐IF, *n* = 15) for 12 weeks (Figure 1). The animals were allocated by a stratified randomization based on the maximum speed reached at maximal exercise capacity test performed at the beginning of the experimental protocol. All groups received commercial rat chow (Nuvilab® CR1, Brazil), and had free access to drinking water. Diet energy density was 3.61 kcal/g; diet consisted of 20.96% protein, 52.28% carbohydrate, and 3.76% fat, according to manufacturer. Additionally, the diet contained 9.60% ash and 13.40% moisture. The C and T groups received *ad libitum* chow daily, while IF and T‐IF received the same chow every other day (alternating with 24‐h total fasting). T and T‐IF animals were subjected to a treadmill running protocol, based on a previous study (Carvalho et al., 2021).

### 2.2 Maximum exercise capacity and high‐intensity interval training protocol

Animals underwent an adaptation period on the treadmill 1 week before the effort test and the HIIT protocol. Following the adaptation period, exercise testing was conducted using an incremental protocol, according to previous studies (Carvalho et al., 2021; Moreira et al., 2013). The exercise testing was performed at four time points: before protocol initiation (M1), four (M2) and eight (M3) weeks post‐protocol initiating, and at the end of the experiment (M4). All animals underwent the test at M1 and M4. Maximum running speed attained at M1 determined initial training load. Only animals from the T and T‐IF groups underwent the M2 and M3 tests, updating training load (Carvalho et al., 2021; Moreira et al., 2013). The training protocol was started after the maximal exercise capacity test. HIIT was performed on a treadmill five times per week for 12 weeks, according to previous studies (Carvalho et al., 2021).

### 2.3 Tissue collection

Rats were anesthetized with intraperitoneal sodium thiopental (100 mg/kg) and euthanized by decapitation. After blood collection, gastrocnemius muscles from the right and left hind limbs were dissected, weighed, separated into samples, frozen in liquid nitrogen, and stored at −80°C. After median laparotomy, white adipose depots from epididymal and retroperitoneal sites were removed and weighed. The right tibia was dissected, measured with a pachymeter, and used to normalize soleus and gastrocnemius muscle mass.

### 2.4 Nutritional profile and body composition

Food consumption was measured daily and body weight (BW) once a week. Calorie intake was calculated as follows: food consumption × diet energy density. Feed efficiency (the ability to convert calorie intake into BW) was determined by dividing BW gain (g) by total calorie intake (Kcal) (Oliveira Junior et al., 2010). Lee index (∛MC/CR, MC = body mass; CR = naso‐anal length of the rat) represents the cubic root of the ratio between rat body mass and naso‐anal length (Lee, 1929). Adiposity index (AD) was calculated from sum of the weights from individual fat sites: Σfat sites × 100/body weight (Oliveira‐Junior et al., 2019).

### 2.5 Serum biochemical profile

Blood was collected; serum was separated by centrifugation at 3000×*g* for 10 min and then stored at −80°C for subsequent assessment. Glucose, total cholesterol, triacylglycerol, albumin, and total protein serum levels were measured by spectrophotometry using enzymatic kits (Kovalent Diagnosis, Rio de Janeiro, RJ, Brazil) (Oliveira Junior et al., 2010).

### 2.6 Morphological analysis

Skeletal muscle macroscopic morphology was analyzed by assessing the following variables: soleus muscle mass, gastrocnemius muscle mass, and the ratios between mass of soleus and gastrocnemius muscles and length of the tibia (soleus/tibia and gastrocnemius/tibia) (Basilio et al., 2020).

Microscopic morphological analysis used histological cross‐sections (thickness of 10 μm) cut from fragments of the soleus muscles and the white (superficial) portion of the gastrocnemius muscle in a cryostat at −20°C. Histological sections were stained with the hematoxylin and eosin technique and used to measure the cross‐sectional fiber area from 150 to 200 fibers per animal (Martinez et al., 2016). Histological sections were analyzed at 400× magnification using a Leica DM5500B microscope (Wetzlar, Germany) coupled to a digital image projection video camera equipped with Leica Application Suite version 4.0.0 (Heerbrugg, Switzerland). Muscle fiber area was measured using *ImageJ* software (Wayne Rasbandat NIH, USA).

### 2.7 Western blot

Protein levels were analyzed by Western blot (Carvalho et al., 2021; Martinez et al., 2016) using primary antibodies for the following target proteins: ARC (rabbit polyclonal antibody H‐150, sc‐11435, Santa Cruz Biotechnology, Inc., CA, USA) AIF (mouse monoclonal antibody E‐1, sc‐13116, Santa Cruz Biotechnology, Inc., CA, USA); EndoG (mouse monoclonal antibody B‐2, sc‐365359, Santa Cruz Biotechnology, Inc., CA, USA); poly (ADP‐ribose) polymerase 1 (PARP‐1) (rabbit polyclonal antibody H‐250, sc‐7150, Santa Cruz Biotechnology, Inc., CA, USA); myostatin (rabbit polyclonal antibody GDF‐8 (N‐19), sc‐6885R, Santa Cruz Biotechnology, Inc., CA, USA). Protein levels were normalized to those of glyceraldehyde‐3‐phosphate dehydrogenase (GAPDH) (mouse monoclonal antibody 6C5, sc‐32233, Santa Cruz Biotechnology, Inc., CA, USA). Briefly, protein was extracted from soleus and gastrocnemius fragments using HEPES buffer (1 mL/100 mg tissue) containing protease and phosphatase inhibitors. Supernatant protein content was quantified by Bradford assay. Samples were separated on a polyacrylamide gel and then transferred to a nitrocellulose membrane. Blotted membrane was stained with 0.1% Ponceau S solution (in 5% acetic acid, purchased from Sigma‐Aldrich, St. Louis, MO, USA) for monitoring equal loading of samples and transfer efficiency. After blockade with 5% skimmed milk in TBST for 1 h at room temperature, membrane was incubated with the primary antibodies (overnight at 4°C). Membrane was then washed with TBST and incubated with secondary peroxidase‐conjugated antibodies using monoclonal mouse anti‐rabbit IgG‐HRP antibody (sc‐2357, Santa Cruz Biotechnology, Inc., CA, USA) or anti‐mouse m‐IgG kappa light chain binding protein‐HRP (sc‐516102, Santa Cruz Biotechnology, Inc., CA, USA) (90 min at room temperature). Super Signal® West Pico Chemiluminescent Substrate (Pierce Protein Research Products, Rockford, USA) was used to detect bound antibodies by autoradiography. After image acquisition, the membrane was soaked with ReBlot Plus Strong Antibody Stripping Solution, 10× (Millipore, Burlington, Massachusetts, USA) to remove antibodies attached to the membrane. Then, the blockade process was performed again, and the membrane incubated overnight at 4°C with GAPDH primary antibody. This procedure was repeated until the signal was detected and the autoradiography obtained. Films were scanned and bands quantified by densitometry using a Gel‐Pro Analyzer 3.1 (Media Cybernetics, Silver Spring, MD, USA).

### 2.8 Statistical analysis

Results are expressed as descriptive measures of centralization and variability. Data distribution was analyzed using the Kolmogorov–Smirnov test. Variables were evaluated by two‐way analysis of variance (Two‐Way ANOVA). When significant differences were found (*p* < 0.05), a post hoc Tukey's multiple comparisons test was performed (comparisons of interest: C vs. IF, C vs. T, IF vs. T‐IF, T vs. T‐IF). Significance level was set at 5%.

## 3 RESULTS

**FIGURE 2:**
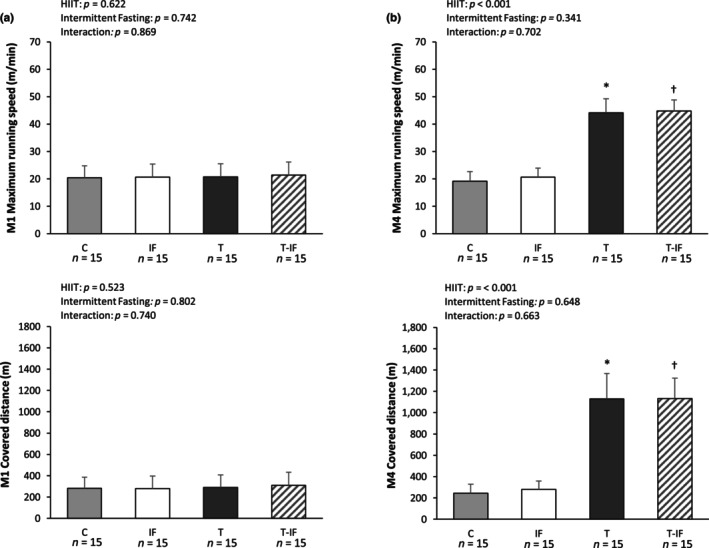
Maximal running speed (a) and covered distance (b) reached at maximal exercise performance in the treadmill running test, before (M1) and after (M4) training protocol. C, control; IF, intermittent fasting; T, high‐intensity interval training; T‐IF, high‐intensity interval training combined with intermittent fasting. Two‐way ANOVA and Tukey test; *p < 0.05 versus C, †*p* < 0.05 versus IF.

At the beginning of the experiment (M1), maximum running speed (C 20 ± 4; IF 21 ± 5; T 21 ± 5; T‐IF 21 ± 5 m/min) and covered distance (C 282 ± 102; IF 280 ± 116; T 291 ± 117; T‐IF 309 ± 123 m) at maximal exercise tolerance test were similar between the groups. At the end of the protocol (M4), both maximum running speed (C 19 ± 4; IF 21 ± 3; T 44 ± 5; T‐IF 45 ± 4 m/min) and covered distance (C 243 ± 85; IF 280 ± 80; T 1131 ± 237; T‐IF 1132 ± 192 m) were higher in T than C and higher in T‐IF than IF (Figure 2).

Nutritional profile and body composition are presented in Table 1. Total food intake, total calorie intake, and total food efficiency were lower in IF and T than C, and lower in T‐IF than T. Food efficiency was lower in T‐IF than IF. Final body weight and Lee index were lower in IF and T‐IF than in their respective controls (C and T); additionally, body weight was lower in T than C. AD was lower in T than C and lower in T‐IF than IF and T. Epididymal and retroperitoneal fat depot masses were lower in IF and T than C, and lower in T‐IF than IF and T.

**TABLE 1:** Nutritional profile and body composition.

Variable	Groups				Factors (*p*‐value)		
	C (*n* = 15)	IF (*n* = 15)	T (*n* = 15)	T‐IF (*n* = 15)	HIIT	Intermittent fasting	Interaction
Total food intake (g)	2147 ± 60	1586 ± 48*	2079 ± 68*	1615 ± 84**	0.258	<0.001	0.007
Total caloric intake (Kcal)	7751 ± 218	5727 ± 175*	7505 ± 248*	5830 ± 304**	0.258	<0.001	0.007
Total food efficiency (%)	3.3 ± 0.3	3.0 ± 0.3*	2.9 ± 0.2*	2.6 ± 0.3**, ***	<0.001	<0.001	0.818
Final body weight (g)	439 ± 52	363 ± 38*	406 ± 35*	346 ± 21**	0.016	<0.001	0.438
Lee index (g/cm^3^)	0.29 ± 0.01	0.27 ± 0.01*	0.28 ± 0.01	0.27 ± 0.01**	0.048	<0.001	0.578
Adiposity (%)	3.5 ± 0.7	3.1 ± 0.9	2.5 ± 0.5*	1.7 ± 0.4**, ***	<0.001	<0.001	0.289
Epididymal fat (g)	9.3 ± 2.4	6.9 ± 2.1*	6.5 ± 1.7*	4.2 ± 1.1**, ***	<0.001	<0.001	0.973
Retroperitoneal fat (g)	6.4 ± 2.3	4.2 ± 1.9*	3.8 ± 1.2*	1.6 ± 0.6**, ***	<0.001	<0.001	0.937

*Note*: Mean ± SD. Two‐way ANOVA and Tukey test.

Abbreviations: C, sedentary control; IF, intermittent fasting; T, high‐intensity interval training; T‐IF, high‐intensity interval training and intermittent fasting.

* *p* < 0.05 versus C.

** *p* < 0.05 versus T.

*** *p* < 0.05 versus IF.

Glucose, total cholesterol, and serum albumin concentrations did not differ between the groups. Triglycerides serum concentration was lower in T than in C and T‐IF (Table 2).

**TABLE 2:** Biochemical profile.

Variable	Groups				Factors (*p*‐value)		
	C (*n* = 15)	IF (*n* = 15)	T (*n* = 15)	T‐IF (*n* = 15)	HIIT	Intermittent fasting	Interaction
Glucose (mg/dL)	122 ± 26	115 ± 38	110 ± 11	104 ± 34	0.142	0.406	0.919
Cholesterol (mg/dL)	79 ± 10	74 ± 10	74 ± 9	75 ± 11	0.448	0.411	0.267
HDL (mg/dL)	49 ± 8	43 ± 7	49 ± 8	47 ± 7	0.442	0.079	0.267
Non‐HDL (mg/dL)	29 ± 7	30 ± 9	25 ± 7	28 ± 8	0.152	0.266	0.674
Triglycerides (mg/dL)	84 ± 20	77 ± 9	69 ± 11*	79 ± 11**	0.064	0.577	0.024
Albumin (g/dL)	3.3 ± 0.5	3.5 ± 0.5	3.2 ± 0.3	3.4 ± 0.4	0.227	1.000	0.740

*Note*: Mean ± SD. Two‐way ANOVA and Tukey test.

Abbreviations: C, sedentary control; IF, intermittent fasting; T, high‐intensity interval training; T‐IF, high‐intensity interval training and intermittent fasting.

* *p* < 0.05 versus C.

** *p* < 0.05 versus T.

Soleus muscle weight and soleus weight‐to‐tibia length ratio did not differ between the groups. Gastrocnemius muscle weight and gastrocnemius weight‐to‐tibia length ratio were lower in IF and T‐IF than in C and T, respectively. Gastrocnemius muscle mass was lower in T than C (Table 3).

**TABLE 3:** Macroscopic muscle morphology.

Variable	Groups				Factors (*p*‐value)		
	C (*n* = 15)	IF (*n* = 15)	T (*n* = 15)	T‐IF (*n* = 15)	HIIT	Intermittent fasting	Interaction
Soleus (g)	0.14 ± 0.02	0.13 ± 0.03	0.14 ± 0.02	0.15 ± 0.02	0.183	0.716	0.575
Soleus/tibia (mg/mm)	3.2 ± 0.5	3.22 ± 0.75	3.3 ± 0.5	3.5 ± 0.6	0.146	0.497	0.644
Gastrocnemius (g)	2.5 ± 0.2	2.14 ± 0.21*	2.3 ± 0.2*	2.1 ± 0.1**	0.058	<0.001	0.318
Gastrocnemius/tíbia (mg/mm)	58 ± 4.7	50 ± 4.5*	55 ± 4.9	49 ± 3.6**	0.061	<0.001	0.400

*Note*: Mean ± SD. Two‐way ANOVA and Tukey test.

Abbreviations: C, sedentary control; IF, intermittent fasting; T, high‐intensity interval training; T‐IF, high‐intensity interval training and intermittent fasting.

* *p* < 0.05 versus C.

** *p* < 0.05 versus T.

**FIGURE 3:**
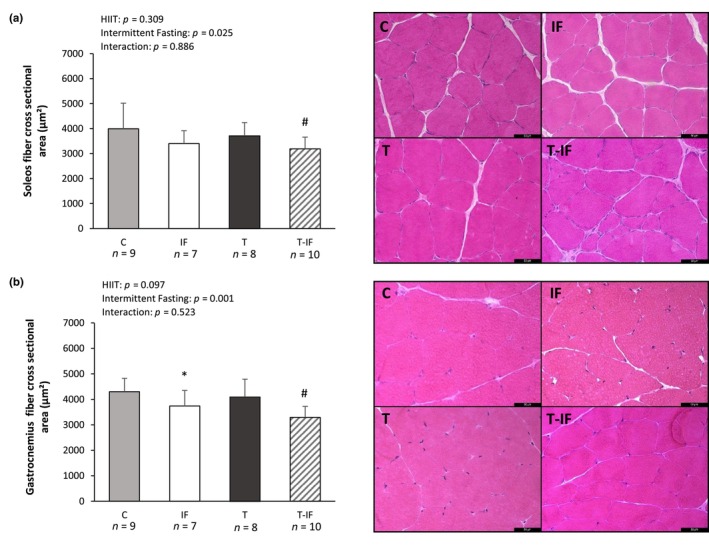
Cross‐sectional area and representative transverse histological sections of the soleus (a) and gastrocnemius (b) muscles (400× magnification) stained with hematoxylin–eosin. T, high‐intensity interval training; T‐IF, high‐intensity interval training combined with intermittent fasting. Values expressed as mean ± SD. Two‐way ANOVA and Tukey test; *p < 0.05 versus C, #*p* < 0.05 versus T.

Cross‐sectional fiber area in soleus (C 3991 ± 1024; IF 3402 ± 513; T 3714 ± 526; T‐IF 3192 ± 465 μm^2^) and gastrocnemius (white portion; C 4301 ± 521; IF 3742 ± 610; T 4098 ± 689; T‐IF 3292 ± 431 μm^2^) muscles were lower in T‐IF than T. Gastrocnemius cross‐sectional fiber area was lower in IF than C (Figure 3).

**FIGURE 4:**
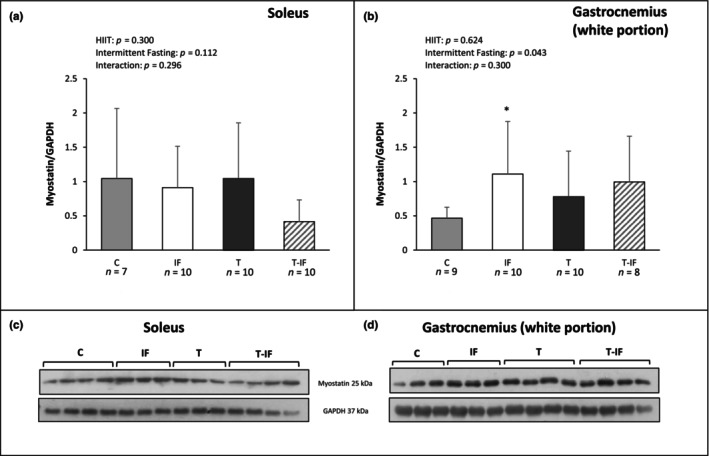
Western blot protein levels of myostatin in (a) soleus muscle and (b) gastrocnemius muscle (white portion); Protein levels were normalized to those of glyceraldehyde‐3‐phosphate dehydrogenase (GAPDH). Western blot representative protein expression in (c) soleus muscle and (d) gastrocnemius muscle (white portion). Myostatin; C, control; IF, intermittent fasting; T, high‐intensity interval training; T‐IF, high‐intensity interval training combined with intermittent fasting. Values expressed as mean ± SD. Two‐way ANOVA and Tukey test; *p < 0.05 versus C.

Myostatin expression did not differ between the groups in soleus muscle (C 1.04 ± 1.02; IF 0.91 ± 0.60; T 1.05 ± 0.80; T‐IF 0.41 ± 0.31 arbitrary units) and was higher in IF than C in gastrocnemius muscle white portion (C 0.46 ± 0.16; IF 1.11 ± 0.76; T 0.78 ± 0.66; T‐IF 1.00 ± 0.66 arbitrary units; Figure 4).

**FIGURE 5:**
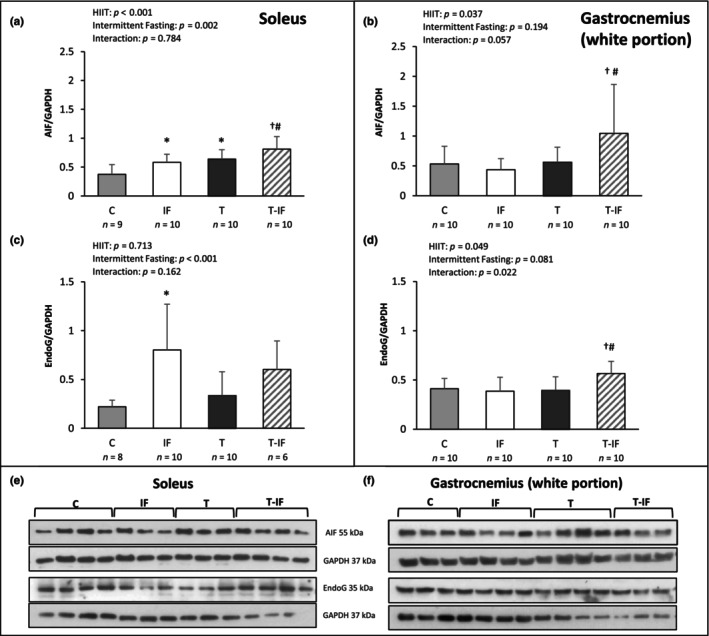
Western blot protein levels Apoptosis‐inducing factor (AIF) in (a) soleus muscle and (b) gastrocnemius muscle (white portion); endonuclease G (EndoG) in (c) soleus muscle and (d) gastrocnemius muscle (white portion). Protein levels were normalized to those of glyceraldehyde‐3‐phosphate dehydrogenase (GAPDH). Western blot representative protein expression in (e) soleus muscle and (f) gastrocnemius muscle (white portion) AIF; EndoG; C, control; IF, intermittent fasting; T, high‐intensity interval training; T‐IF, high‐intensity interval training combined with intermittent fasting. Values expressed as mean ± SD. Two‐way ANOVA and Tukey test; *p < 0.05 versus C; #*p* < 0.05 versus T; †*p* < 0.05 versus IF.

Concerning apoptosis signaling proteins (Figure 4): AIF protein expression was higher in IF and T than C, and higher in T‐IF than T and IF in soleus muscle (C 0.38 ± 0.16; IF 0.58 ± 0.13; T 0.63 ± 0.16; T‐IF 0.81 ± 0.21 arbitrary units); in gastrocnemius muscle white portion, AIF (C 0.53 ± 0.29; IF 0.44 ± 0.18; T 0.56 ± 0.25; T‐IF 1.04 ± 0.82 arbitrary units) was higher in T‐IF than T and IF. Endo‐G expression was higher in IF than C in soleus muscle (C 0.22 ± 0.06; IF 0.80 ± 0.46; T 0.34 ± 0.24; T‐IF 0.60 ± 0.29 arbitrary units), and higher in T‐IF than T and IF in gastrocnemius muscle white portion (C 0.41 ± 0.10; IF 0.39 ± 0.14; T 0.40 ± 0.13; T‐IF 0.56 ± 0.12 arbitrary units; Figure 5).

**FIGURE 6:**
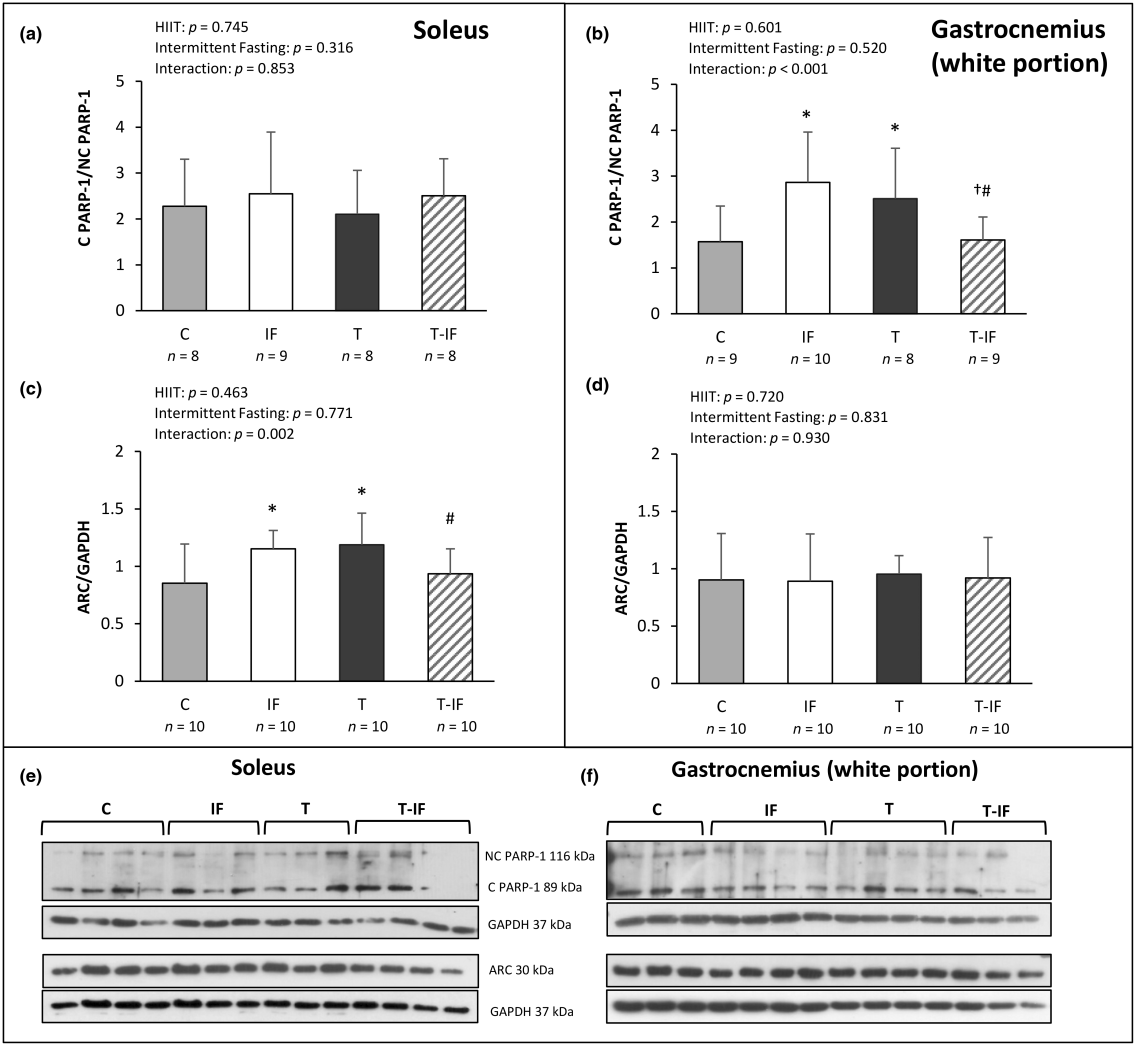
Western blot protein levels of non‐cleaved poly (ADP‐ribose) polymerase 1 (NC PARP‐1); cleaved poly (ADP‐ribose) polymerase 1 (C PARP‐1) in (a) soleus muscle and (b) gastrocnemius muscle (white portion). Apoptosis repressor with caspase recruitment domain (ARC) in (c) soleus muscle and (d) gastrocnemius muscle (white portion). Protein levels were normalized to those of glyceraldehyde‐3‐phosphate dehydrogenase (GAPDH). Western blot representative protein expression in (e) soleus muscle and (f) gastrocnemius muscle (white portion). non‐cleaved poly (ADP‐ribose) polymerase 1 (NC PARP‐1); cleaved poly (ADP‐ribose) polymerase 1 (C PARP‐1); apoptosis repressor with caspase recruitment domain (ARC). C, control; IF, intermittent fasting; T, high‐intensity interval training; T‐IF, high‐intensity interval training combined with intermittent fasting. Values expressed as mean ± SD. Two‐way ANOVA and Tukey test; *p < 0.05 versus C; #*p* < 0.05 versus T; †*p* < 0.05 versus IF.

Cleaved‐to‐non‐cleaved PARP‐1 ratio did not differ between the groups in soleus (C 2.28 ± 1.02; IF 2.55 ± 1.34; T 2.10 ± 0.95; T‐IF 2.50 ± 0.80 arbitrary units), was higher in T and IF than C, and lower in T‐IF than in T and IF in white portion gastrocnemius muscle (C 1.57 ± 0.77; IF 2.87 ± 1.09; T 2.51 ± 1.10; T‐IF 1.61 ± 0.50 arbitrary units; Figure 5). ARC expression was higher in T and IF than in C, and lower in T‐IF than T in soleus muscle (C 0.85 ± 0.34; IF 1.15 ± 0.16; T 1.18 ± 0.27; T‐IF 0.94 ± 0.21 arbitrary units); in gastrocnemius muscle white portion, ARC did not differ between the groups (C 0.90 ± 0.40; IF 0.90 ± 0.41; T 0.95 ± 0.16; T‐ IF 0.92 ± 0.35 arbitrary units; Figure 6).

## 4 DISCUSSION

In this study, we evaluated the influence of combined IF and HIIT on the signaling pathways involved in skeletal muscle apoptosis and atrophy in healthy rats. IF, as an isolated factor, reduced gastrocnemius muscle mass and increased the expression of pro‐apoptotic proteins AIF and EndoG in the soleus muscle; it also increased cleaved‐to‐non‐cleaved PARP‐1 ratio and myostatin expression in gastrocnemius white portion. HIIT alone had no influence on muscle mass or fiber cross‐sectional area in soleus and gastrocnemius white portion muscles; however, HIIT increased AIF and ARC expression in the soleus and increased the cleaved‐to‐non‐cleaved PARP‐1 ratio in gastrocnemius. The combination of IF and HIIT resulted in specific changes, such as increased EndoG and AIF expression and decreased cleaved‐to‐non‐cleaved PARP‐1 ratio in gastrocnemius muscle white portion. Furthermore, combined IF and HIIT increased AIF and reduced ARC expression in soleus muscle. Additionally, fiber cross‐sectional area was reduced in both muscles subjected to the combined interventions. These results suggest that combined IF and HIIT significantly impacted the molecular and morphological characteristics of soleus and gastrocnemius (white portion) muscles.

Animals subjected to combined IF and HIIT had lower body mass, adiposity index, and total food intake. There is evidence that IF‐induced changes in the central nervous system are associated with the release of hormones responsible for hunger and satiety (Hoddy et al., 2016; Lemmens et al., 2011). Additionally, high‐intensity exercise increases energy expenditure and maximum oxygen consumption (Boukabous et al., 2019), as well as improves fat mobilization and consumption, contributing to body mass reduction (Alkahtani et al., 2013). Therefore, our study showed that the combination of HIIT and IF was more effective than these interventions alone in reducing body fat mass in rats.

Concerning biochemical serum profile, physical training alone was more effective than IF in decreasing triglyceride level, a health predictor. However, combined HIIT and IF resulted in a higher triglyceride concentration than in the T group, but IF and T‐IF groups had similar values for this variable. IF improves food efficiency (Cechowska‐Pasko et al., 2004; Varady et al., 2013), which contributes to reduce the use of energy substrate and consequently increases triglyceride availability in the blood. On the other hand, HIIT increases energy expenditure and maximum oxygen consumption (Boukabous et al., 2019) and improves fat mobilization and use (Alkahtani et al., 2013), which could explain the reduction in circulating triglyceride levels. Therefore, it is possible that the combination of IF and HIIT reduced the use of triglycerides and consequently increased their availability in the blood (Elashry et al., 2017; Koskelo et al., 1990).

In our study, HIIT increased the rat's tolerance to exertion, as seen by the higher maximum speed and distance covered during the maximal treadmill test. The increase in exercise tolerance is related to increased oxidative capacity, as HIIT and endurance exercises enhance mitochondrial function and aerobic capacity of skeletal muscles (Hoshino et al., 2016). Although specific oxidative markers were not evaluated in this study, we can speculate that the improvement in exercise tolerance reflects enhanced mitochondrial biogenesis, function, and oxidative capacity in skeletal muscle (Cox et al., 2010; Proctor et al., 1995). In this study, increased exercise tolerance was also observed in the animals submitted to combined HIIT and IF, despite the reduced cross‐sectional area (CSA) of the soleus and gastrocnemius muscle fibers. Even though IF contributed to the reduced fiber CSA, it apparently had no negative impact on functional capacity, either alone or in combination with HIIT. The increase in oxidative capacity may lead to smaller muscle fibers, as the fiber type‐fiber size paradox suggests that despite their high protein synthesis capacity, highly oxidative fibers remain relatively small (van Wessel et al., 2010), showing that reduction in fiber CSA does not always result in muscle weakness or impairment.

The white portion of the gastrocnemius muscle is predominantly composed of fast‐twitch (glycolytic) fibers, which are more susceptible to CSA reduction induced by caloric restriction and endurance exercise (Deschenes et al., 1995; Elashry et al., 2017; Faitg et al., 2019). In our study, the lack of a protective effect on muscle mass or CSA may be attributed, at least in part, to the increased oxidative demand induced by HIIT, analogously to what is observed following endurance exercise (Hoshino et al., 2016). The improvement in mitochondrial function and aerobic capacity may result in potentially smaller yet more efficient muscle fibers (van Wessel et al., 2010), as mentioned above. Alternatively, although HIIT intensely recruits these fast‐twitch fibers due to high‐intensity bouts (Eigendorf et al., 2018), combining HIIT with a fasting state may be associated with a reduced ATP availability and consequently an increased protein catabolism in type II fibers. This may explain, at least partially, why the training was not effective in attenuating the IF‐induced muscle mass and CSA reduction.

Furthermore, it is possible that the different muscle responses are more related to general recruitment than to fiber type composition, since soleus is a highly oxidative postural and constantly activated muscle. Analyzing a muscle with fast oxidative glycolytic fibers, such as the red gastrocnemius, which is quite different from the soleus (slow oxidative) or the white gastrocnemius (fast glycolytic), may shed more light on the effect of fiber type on responses to IF and IF plus training and should be included in future studies.

Concerning the signaling pathways involved in muscle atrophy, studies indicate that myostatin activation is involved in the caloric restriction‐induced decrease in muscle mass (Matsakas et al., 2013). In this study, IF increased myostatin expression in the gastrocnemius muscle white portion indicating a muscle‐specific response to nutrient shortage. Elashry et al. 2017 suggested that fast‐twitch muscle fibers are more prone to catabolic signaling during calorie restriction. This concept is supported by the lack of significant changes in soleus myostatin expression in response to IF and HIIT. Myostatin expression can be influenced by differences in muscle fiber composition, metabolic characteristics (Tobias & Galpin, 2020), or basal myostatin levels (Carlson et al., 1999).

On the other hand, HIIT can inhibit myostatin. Biglari et al. 2020 observed inhibitory effects of HIIT in rats subjected to 30 min of training three times a week. Indeed, HIIT prevented the increase in myostatin induced by IF in the white portion of the gastrocnemius. However, we did not observe an isolated effect of HIIT, which can be related to the higher volume of the HIIT protocol used in this study (Sabag et al., 2022).

The findings on myostatin modulation highlight a complex regulatory mechanism in muscle response to IF and HIIT, suggesting a potential involvement of other signaling pathways. We next evaluated apoptosis signaling‐related proteins from the caspase‐independent pathway and found that combined IF and HIIT increased AIF and EndoG expression in both soleus and gastrocnemius muscles. IF and HIIT are both related to reduced ATP availability, which possibly leads to activation of calpains (Pal et al., 2001). These enzymes can trigger sarcoplasmic reticulum stress, modulating mitochondrial function (Li et al., 2001) and inducing the release of pro‐apoptotic proteins such as AIF (Daugas et al., 2000) and EndoG that translocate to the nucleus and induce DNA fragmentation and apoptosis signaling (Li et al., 2001). We observed increased AIF and EndoG expression due to IF and HIIT, either alone or in combination which can be justified by the high demand for energy expenditure and/or decreased food consumption, favoring ATP reduction. The increased expression of AIF and EndoG caused by combined IF and HIIT may partially explain the absence of any protective effects of training on fasting‐induced muscle loss.

In contrast, we observed that IF and HIIT individually increased cleaved‐to‐non‐cleaved PARP‐1 ratio in gastrocnemius muscle white portion; however, when combined they reduced this ratio. Poly (ADP‐ribose) polymerase‐1 (PARP‐1) is a constitutive nuclear and mitochondrial enzyme that plays a fundamental role in cellular responses to stress (Kraus, 2008). Located at the core of cellular stress pathways, PARP‐1 processes diverse signals acting as a stress sensor and mediator, guiding cells toward specific outcomes (such as DNA repair or cell death) based on the nature and intensity of the stressor. PARP‐1 uses NAD+ as an ADP‐ribose unit donor; therefore, PARP‐1 activity is closely linked to the nuclear metabolism of NAD+ and the general metabolic profile of the cell. In isolated interventions, the increased cleaved‐to‐non‐cleaved PARP‐1 ratio points to cellular adaptation induced by exercise, which increases DNA repair and resistance against oxidative stress in proteins, as well as the protective effect of IF. However, the combined IF and HIIT increased the stress level, potentially generating competition for NAD+ (Gissel & Clausen, 2001; Ryu et al., 2018), and consequently reducing PARP‐1 cleavage.

In relation to antiapoptotic signaling, ARC expression did not differ in gastrocnemius, but was increased in IF and T groups in soleus muscle. ARC is an antiapoptotic protein capable of inhibiting apoptosis mediated by the death receptor and the mitochondrial pathway (Koseki et al., 1998; Nam et al., 2004). Interestingly, ARC expression is high in long‐lived cells such as cardiac and skeletal myocytes (Abmayr et al., 2004; Koseki et al., 1998); however, it is not uniformly expressed in muscle (Quadrilatero & Bloemberg, 2010) and is fiber type specific, with a predominance for Type I and IIA fibers (McMillan & Quadrilatero, 2011). Elderly mice with deletion of the ARC gene had a significant reduction in soleus and plantar cross‐sectional area (Vorobej et al., 2018). We found that increased ARC expression was associated with unchanged fiber cross‐sectional area in the soleus muscle of rats subjected to HIIT or IF alone. However, in the T‐IF group, soleus fiber cross‐sectional area was reduced, even with ARC levels similar to those in the IF and T groups. These results suggest that, faced with increased apoptotic signaling, ARC expression was not sufficient to prevent atrophy in the T‐IF group. In addition, it seems that muscle responses to IF and HIIT are directly impacted by fiber type composition (Barnes et al., 2015; McMillan & Quadrilatero, 2011; Zou et al., 2023).

Although the soleus muscle and the white portion of the gastrocnemius muscle were analyzed, evaluating a muscle with fast oxidative glycolytic fibers, such as the red portion of the gastrocnemius muscle, could have provided a more comprehensive understanding of muscle fiber type responses to IF and HIIT; however, we did not include this analysis, and this may be a limitation of our study. In addition, the lack of analysis of classic oxidative markers limited our understanding of metabolic adaptations. Furthermore, future studies should analyze the potential of other signaling pathways, such as AMPK and mTOR, in modulating muscle responses to HIIT and IF. Another limitation is the fact that we only used male rats, which may not allow us to extrapolate our results to female rats.

## 5 CONCLUSION

Combined IF and HIIT reduce fiber cross‐sectional area of soleus and gastrocnemius (white portion) muscles, which is modulated, at least partially, by caspase‐independent apoptosis signaling. IF‐induced muscle mass loss in fast‐twitch muscle is associated with increased myostatin. Molecular muscle responses to IF and HIIT are directly impacted by muscle fiber type composition.

## FUNDING INFORMATION

The study received financial support from the Federal University of Mato Grosso do Sul, UFMS/MEC, Brazil, Coordination for the Improvement of Higher education Personnel, Brazil (CAPES)—Finance Code 001; and National Council for Scientific and Technological Development (CNPq; processes 311588/2022‐0 and 409191/2021‐3); Foundation to Support Education, Science, and Technology Development, Mato Grosso do Sul, Brazil (FUNDECT) and Programa Pesquisa para o SUS: gestão compartilhada em saúde – PPSUS, with financial support from Decit/SCTIE/MS, through CNPq, FUNDECT and SES‐MS (Notice: FUNDECT ‐ PPSUS 08/2020—process: 25/2021). The supporters had no role in study design, data collection and analysis, decision to publish, or manuscript preparation.

## CONFLICT OF INTEREST STATEMENT

None of the authors has any conflicts of interests related to this research.

## ETHICS STATEMENT

The experimental protocol was approved by the Animal Ethics Committee of Federal University of Mato Grosso do Sul, Brazil (Protocol number 995/2018) in accordance with the Brazilian College of Animal Experimentation (COBEA) and the US National Institutes of Health “Guide for the Care and Use of Laboratory Animals” (National Research Council (US) Committee for the Update of the Guide for the Care and Use of Laboratory Animals, 2011).
